# Prevalence of Congenital Hypothyroidism and Efficacy of Current Cord Sample Screening in the Presence of Metabolic Screening: A Retrospective Cohort Study

**DOI:** 10.7759/cureus.79185

**Published:** 2025-02-17

**Authors:** Ismail M Alwadani, Ahad M Almohammadi, Wijdan N Alzamzami

**Affiliations:** 1 Neonatology, Johns Hopkins Aramco Healthcare, Dhahran, SAU; 2 Pediatrics, Johns Hopkins Aramco Healthcare, Dhahran, SAU

**Keywords:** congenital hypothyroidism, cord blood, diagnostic accuracy, metabolic screening, neonatal screening, prevalence, saudi arabia, serum testing, thyroid function

## Abstract

Background and objectives:Congenital hypothyroidism (CH) is a critical condition that can lead to severe neurodevelopmental impairments if not diagnosed and treated promptly. Early detection through neonatal screening is essential. This study evaluated the efficacy of cord blood free thyroxin 4 (CBFT4) screening for detecting CH in comparison to serum sample testing and newborn metabolic screening (NBS). The aim was to determine the efficacy of current cord blood screening for congenital hypothyroidism in the presence of metabolic screening and to establish the prevalence of CH in a single tertiary center in Saudi Arabia.

Methods: This retrospective cohort study was conducted at Johns Hopkins Aramco Healthcare Center and included all newborns born between January 2021 and December 2023. Both cord blood and heel-prick T4 and thyroid-stimulating hormone (TSH) results were collected from the medical records of 3328 newborns. Diagnostic accuracy parameters were calculated for cord blood and serum findings. Demographic and clinical data were analyzed, including maternal risk factors.

Results: The CH prevalence was 0.8%, which was confirmed by NBS, and there was a higher initial detection rate of 3.4% based on cord blood screening. The average level of cord blood total thyroxin 4 (CBTT4) was 6.48 ± 0.78 µg/dL, and the cord blood TSH was 5.9 ± 6.4 µIU/mL. Serum FT4 levels were 1.3 ± 0.5 ng/dL, and serum TSH levels were 8.7 ± 24.5 µIU/mL. The sensitivity and specificity of cord blood diagnosis were 50% and 97%, respectively, while serum sample diagnosis had a sensitivity of 62.5% and a specificity of 98.6%. Significant associations were found between lower birth weight (2.146 ± 0.771 kg vs. 2.801 ± 0.662 kg; *p =* 0.006), lower gestational age (35 ± 3 weeks vs. 37 ± 3 weeks; *p =* 0.008), and CH. Cord blood TSH levels were significantly higher in CH cases (32.9 ± 55.2 µIU/mL vs. 5.7 ± 3.7 µIU/mL; *p <* 0.001).

Conclusion: Cord blood screening for congenital hypothyroidism demonstrates moderate sensitivity and high specificity, indicating that it is reliable for ruling out the condition but necessitates confirmatory serum testing due to the risk of false positives. The prevalence of CH in this cohort aligns with global estimates. Lower birth weight, lower gestational age, and maternal thyroid disease are significant risk factors for CH. A two-tiered screening approach with follow-up serum testing is recommended to improve diagnostic accuracy and ensure timely intervention.

## Introduction

Congenital hypothyroidism (CH) is a significant endocrine disorder that results from insufficient production of thyroid hormones in newborns. This condition can lead to severe and irreversible neurodevelopmental delays if not diagnosed and treated promptly [1,2]. Early detection and treatment of CH are paramount to preventing adverse outcomes. The introduction of neonatal screening programs has significantly improved early diagnosis and has allowed for timely intervention and better long-term outcomes. Screening programs typically measure levels of thyroid-stimulating hormone (TSH) and free thyroxine (T4) in newborns to identify those at risk of CH [2-4]. These programs have become a standard practice in many countries, including Saudi Arabia, where the prevalence of CH is reported to be relatively high compared to global averages [5].

The prevalence of CH varies widely between different populations and geographic regions. Factors that influence the prevalence include genetic predispositions, environmental influences, and iodine intake. The incidence of CH tends to be higher in regions with iodine deficiency [6-8]. Despite efforts to ensure adequate iodine nutrition, a notable prevalence of CH is still reported in Saudi Arabia, which necessitates robust screening protocols to identify and manage affected newborns effectively [5,6].

Neonatal screening for CH can be conducted using various methods, including heel-prick blood sampling (the Guthrie test) and cord blood sampling. The heel-prick test is performed between 48 and 72 hours after birth and involves collecting a small blood sample from the newborn's heel, which is analyzed for TSH and T4 levels [9,10]. This method is widely used and is considered the gold standard for neonatal screening due to its high sensitivity and specificity. However, the timing of the test can sometimes delay the identification and treatment of affected infants [9-11].

Cord blood sampling is an alternative method that involves collecting blood from the umbilical cord immediately after birth. This method allows for earlier detection of CH and could potentially reduce the time to diagnosis and treatment initiation [12]. Cord blood screening can measure total thyroxin (CBTT4) and TSH levels and provides a rapid assessment of thyroid function. Despite its advantages, however, its efficacy and reliability compared to heel-prick testing remain a subject of ongoing research and debate [12,13].

Several studies have explored the diagnostic accuracy of cord blood screening for detecting CH. Research has shown that while cord blood TSH and T4 levels can effectively identify newborns at risk of CH, there are concerns regarding false positives and negatives. The cutoff values for TSH and T4 in cord blood screening can vary and influence the test's sensitivity and specificity. Consequently, confirmatory testing using serum samples is often recommended to ensure accurate diagnosis [12-15].

Studies in Saudi Arabia have reported varying prevalence rates of CH, which highlights the need for continuous monitoring and evaluation of screening practices [5,16]. The effectiveness of current screening methods, particularly in regions with high prevalence, is crucial for optimizing early diagnosis and intervention strategies. Understanding the diagnostic accuracy of cord blood screening in the context of existing metabolic screening protocols could inform policy decisions and clinical practices and ultimately benefit newborns at risk of CH [12,13].

In addition to screening practices, maternal risk factors play a significant role in the incidence of CH. Maternal thyroid disease, diabetes mellitus, and other health conditions can influence thyroid function in newborns. Identifying and managing these risk factors during pregnancy are essential for preventing CH and ensuring optimal neonatal health. The interplay between maternal health and neonatal thyroid function underscores the importance of a comprehensive approach to screening and prevention [17,18].

Despite advancements in screening technologies and protocols, challenges remain in ensuring accurate and timely diagnosis of CH. The variability in screening methods, cutoff values, and diagnostic criteria necessitates ongoing research and standardization efforts. It is also essential for comparative studies to evaluate the efficacy of different screening approaches, such as cord blood versus heel-prick testing, to establish best practices and improve health outcomes for newborns. 

Therefore, the primary objective of this study was to determine the diagnostic accuracy of cord blood free thyroxin 4 (CBFT4) screening for congenital hypothyroidism (sensitivity, specificity, positive predictive value (PPV), and negative predictive value (NPV)). Furthermore, the diagnostic efficacy of cord blood screening was compared with that of heel-prick serum testing and newborn metabolic screening (NBS) for congenital hypothyroidism. The prevalence of CH was also assessed among newborns at a tertiary care center in Saudi Arabia over a three-year period, and maternal and neonatal risk factors were analyzed.

## Materials and methods

This study employed a retrospective cohort design. The analysis included newborns born between January 2021 and December 2023 at a single tertiary care center. The study was conducted at Johns Hopkins Aramco Healthcare Center, a tertiary care hospital located in Dhahran, Saudi Arabia. The study population included all live-born infants delivered at Johns Hopkins Aramco Healthcare Center from January 2021 to December 2023. This period was chosen to ensure a substantial sample size for robust statistical analysis. Medical records of 3328 newborns were reviewed, including data from cord blood and heel-prick T4 and TSH results, along with demographic and clinical information. All infants born at our center were screened for CBTT4. If CBTT4 measurements were below 7.6 μg/dL, then CBTSH was measured on the same sample. In all cases with CBTT4 <7.6 μg/dL or CBTSH >20 mIU/L, another venous sample for FT4 and TSH was obtained. Samples were run daily for CBTT4 and CBTSH using the Alinity immunoassay (Abbott, Abbott Park, IL, USA) with chemiluminescent microparticle immunoassay (CMIA) technology. In addition, all newborns will undergo newborn metabolic screening at 24 hours of age, which includes screening for congenital hypothyroidism by measuring TSH with a normal cut-off of <15.0 µIU/mL. Premature infants routinely have repeated venous FT4 and TSH tested at four weeks of age.

Inclusion and exclusion criteria

Newborns were included in the study if they were born at Johns Hopkins Aramco Healthcare Center within the specified study period and had both cord blood and heel-prick T4 and TSH results available in their medical records. Additionally, newborns were required to have undergone routine NBS to confirm or rule out CH. Newborns who died before specimen collection for newborn screening or had incomplete screening for CH were excluded from the study. Infants with missing or incomplete medical records that did not provide necessary data for the analysis were also excluded. This ensured that only newborns with complete and reliable data were included in the study.

Data collection and analysis

Data were collected using a standardized data collection sheet that was designed to capture all relevant information from the medical records. The sheet included fields for basic demographic variables (sex and date of birth), gestational age, birth weight, maternal thyroid disease, maternal diabetes mellitus (DM), initial cord-blood T4 result, initial heel-prick TSH result, repeated cord-blood TSH result, confirmed diagnosis, and patient remarks. The data collection sheet ensured consistency and completeness in data gathering.

Data management involved the systematic collection, entry, and cleaning of data to ensure accuracy and completeness. Statistical analysis was performed using SPSS version 25.0 (IBM Corp., Armonk, NY, USA). Descriptive statistics were used to summarize the demographic and clinical characteristics of the study population. Frequencies and percentages were calculated for categorical variables, while means and standard deviations were calculated for continuous variables. Accuracy parameters were calculated, including the sensitivity, specificity, PPV, and NPV. Chi-squared tests and t-tests were used to assess associations between variables, and p-values less than 0.05 were considered statistically significant.

Ethical considerations

Ethical approval for the study was obtained from the Institutional Review Board of Johns Hopkins Aramco Healthcare Center (approval 23-38). The study adhered to the principles of the Declaration of Helsinki and ensured the confidentiality and anonymity of patient data. The study's findings were intended to improve clinical practice and patient outcomes in alignment with ethical guidelines for research involving human subjects.

__PRESENT

## Results

Characteristics of included newborns and maternal risk factors

The study included a total of 1,012 newborns, the sex distribution indicated a higher proportion of males at 58.4% (591 newborns) than females at 41.6% (421 newborns). The mean birth weight was 2.796 ± 0.665 kg (mean ± standard deviation). The gestational age at birth averaged 37 ± 3 weeks. Regarding maturity levels, 0.6% (6 newborns) were extremely premature, 5.8% (59 newborns) were very premature, 20.4% (206 newborns) were late preterm, 34.9% (353 newborns) were early term, 33.5% (339 newborns) were full term, and 4.8% (49 newborns) were late term.

Regarding maternal health factors, 92% (931 mothers) did not have thyroid disease, whereas 8% (81 mothers) had maternal thyroid disease. Furthermore, 12.3% (124 mothers) had gestational diabetes mellitus (GDM), 0.4% (4 mothers) had type 1 diabetes mellitus (T1DM), 0.6% (6 mothers) had type 2 diabetes mellitus (T2DM), and 86.8% (878 mothers) did not have any form of diabetes (Table 1).

**Table 1:** Characteristics of included newborns and maternal risk factors (n=1012). GDM: gestational diabetes mellitus; T1DM: type 1 diabetes mellitus; T2DM: type 2 diabetes mellitus

Parameter		Frequency (%)
Sex	Female	421 (41.6%)
	Male	591 (58.4%)
Weight, kg		2.796 ± 0.665
Gestational age, weeks		37 ± 3
Maturity	Extremely premature	6 (0.6%)
	Very premature	59 (5.8%)
	Late preterm	206 (20.4%)
	Early term	353 (34.9%)
	Full term	339 (33.5%)
	Late term	49 (4.8%)
Maternal thyroid disease	No	931 (92%)
	Yes	81 (8%)
Maternal diabetes mellitus	No	878 (86.8%)
	GDM	124 (12.3%)
	T1DM	4 (0.4%)
	T2DM	6 (0.6%)

Cord blood and serum laboratory results, and NBS confirmatory findings

The laboratory results of the cord blood and serum samples showed that the mean CBTT4 level was 6.48 ± 0.78 µg/dL. The mean cord blood TSH was 5.9 ± 6.4 µIU/mL. Based on the cord blood diagnosis, 34 of the newborns (3.4%) were diagnosed with congenital hypothyroidism, while the remaining 978 (96.6%) were not. The mean serum level of FT4 was 1.3 ± 0.5 ng/dL, whereas the average serum level of TSH was 8.7 ± 24.5 µIU/mL. The serum samples confirmed a CH diagnosis in 19 of the newborns (1.9%) and a lack of CH in 993 newborns (98.1%).

According to the NBS confirmatory diagnosis, eight of the newborns (0.8%) were confirmed to have CH, while 1,004 (99.2%) did not (Table 2). These results indicate that the prevalence of CH detected through cord blood screening was higher than that indicated by serum sample diagnosis and NBS confirmatory testing. This discrepancy underscores the potential limitations of cord blood screening and suggests that while it may serve as an initial screening tool, confirmatory testing via serum samples and NBS is crucial for accurate diagnosis.

**Table 2:** Cord blood and serum laboratory results and NBS confirmatory findings (n=1012). TSH: thyroid-stimulating hormone; FT4: free thyroxine

Parameter		Frequency (%)
Cord blood total thyroxin 4 (CBTT4) (ug/dL)		6.48 ± 0.78
Cord blood TSH (μIU/ml)		5.9 ± 6.4
Cord blood diagnosis	Congenital hypothyroidism	34 (3.4%)
	Normal	978 (96.6%)
Serum FT4		1.3 ± 0.5
Serum TSH		8.7 ± 24.5
Serum sample diagnosis	Congenital hypothyroidism	19 (1.9%)
	Normal	993 (98.1%)
Newborn Metabolic Screen (NBS) Confirmatory diagnosis	Congenital hypothyroidism	8 (0.8%)
	Normal	1004 (99.2%)

Association of NBS confirmatory diagnosis with newborn characteristics and maternal risk factors

The association between various parameters and the NBS confirmatory diagnosis for CH was evaluated. The sex distribution showed that five out of 421 females (1.2%) and three out of 591 males (0.5%) had CH (χ² = 1.45, p = 0.22).

The mean birth weight of newborns diagnosed with CH was significantly lower at 2.146 kg compared to 2.801 kg for those without CH (t = 7.738, p = 0.006). Similarly, the gestational age was significantly lower in the CH group (35 weeks) compared to the group without CH (37 weeks) (t = 7.148, p = 0.008). Extremely premature and late-term newborns showed no cases of CH, whereas one out of 59 very premature (1.7%), five out of 206 late-preterm (2.4%), one out of 353 early-term (0.3%), and one out of 339 of full-term newborns (0.3%) had CH (χ² = 10.309, p = 0.067). Maternal health factors showed that six out of 931 newborns whose mothers did not have thyroid disease were diagnosed with CH (0.6%), whereas two out of 81 newborns whose mothers had thyroid disease (2.5%) had CH (χ² = 3.163, p = 0.075). There were no cases of CH among newborns whose mothers had GDM, T1DM, or T2DM, while eight out of 878 newborns who had mothers without DM (0.9%) were diagnosed with CH (χ² = 1.231, p = 0.746).

Laboratory results showed that the mean CBTT4 level was slightly lower in the CH group (6.07 µg/dL) compared to the group without CH (6.48 µg/dL), but the difference was not statistically significant (t = 2.179, p = 0.140). However, the mean cord blood TSH level was significantly higher in the CH group (32.9 µIU/mL) compared to the group without CH (5.7 µIU/mL) (t = 167.7, p < 0.001). Serum FT4 levels were significantly lower in the CH group (0.8 µg/dL) compared to the group without CH (1.3 µg/dL) (t = 5.52, p = 0.020), and serum TSH levels were significantly higher in the CH group (96.5 µIU/mL) compared to the group without CH (5.7 µIU/mL) (t = 141.9, p < 0.001) (Table 3).

**Table 3:** NBS-confirmed diagnosis in association with characters and risk factors. χ²: Chi-squared test, t: t-test, TSH: thyroid-stimulating hormone; FT4: free thyroxine, GDM: gestational diabetes mellitus, T1DM: type 1 diabetes mellitus, T2DM: type 2 diabetes mellitus

Parameter		Newborn Metabolic Screen (NBS) Confirmatory diagnosis		Test statistic	p-value
		Hypothyroidism	Normal		
Sex	Female	5 (1.2%)	416 (98.8%)	χ² = 1.45	0.229
	Male	3 (0.5%)	588 (99.5%)		
Weight, kg		2.146 ± 0.771	2.801 ± 0.662	t = 7.738	0.006
Gestational age, weeks		35 ± 3	37 ± 3	t = 7.148	0.008
Maturity	Extremely premature	0 (0%)	6 (100%)	χ² = 10.309	0.067
	Very premature	1 (1.7%)	58 (98.3%)		
	Late preterm	5 (2.4%)	201 (97.6%)		
	Early term	1 (0.3%)	352 (99.7%)		
	Full term	1 (0.3%)	338 (99.7%)		
	Late term	0 (0%)	49 (100%)		
Maternal thyroid disease	No	6 (0.6%)	925 (99.4%)	χ² = 3.163	0.075
	Yes	2 (2.5%)	79 (97.5%)		
Maternal diabetes mellitus	No	8 (0.9%)	870 (99.1%)	χ² = 1.231	0.746
	GDM	0 (0%)	124 (100%)		
	T1DM	0 (0%)	4 (100%)		
	T2DM	0 (0%)	6 (100%)		
Cord blood total thyroxin 4 (CBTT4) (μg/dL)		6.07 ± 1.03	6.48 ± 0.78	t = 2.179	0.140
Cord blood TSH (μIU/ml)		32.9 ± 55.2	5.7 ± 3.7	t = 167.7	0.000
Serum FT4 (μg/dL)		0.8 ± 0.1	1.3 ± 0.5	t = 5.52	0.020
Serum TSH (μIU/ml)		96.5 ± 103.8	5.7 ± 6.2	t = 141.9	0.000

Diagnostic test accuracy of cord blood and serum findings

The diagnostic accuracy of cord blood and serum sample findings was evaluated in association with the NBS confirmatory diagnosis. For cord blood diagnosis, CH was identified in four out of 34 newborns (11.8%) with confirmed CH, while 30 out of 34 newborns (88.2%) had false positives. Among those with normal cord blood results, four out of 978 results (0.4%) were false negatives, and 974 out of 978 results (99.6%) were true negatives. The sensitivity of cord blood diagnosis was 50%, the specificity was 97%, the PPV was 11.8%, and the NPV was 99.6%.

Regarding serum sample diagnosis, five out of 19 results (26.3%) with confirmed CH were true positives, while 14 out of 19 results (73.7%) were false positives. Among those with normal serum results, three out of 993 results (0.3%) were false negatives, and 990 out of 993 results (99.7%) were true negatives. The sensitivity of serum diagnosis was 62.5%, the specificity was 98.6%, the PPV was 26.3%, and the NPV was 99.7% (Table 4). These findings suggest that while both cord blood and serum sample screenings are valuable, serum sample diagnosis demonstrated slightly higher sensitivity and specificity compared to cord blood diagnosis. The NPV was particularly high for both methods, indicating their reliability in ruling out CH.

**Table 4:** NBS-confirmed diagnosis in association with cord blood and serum sample diagnoses.

Parameter		Newborn Metabolic Screen (NBS) confirmatory diagnosis	
		Congenital hypothyroidism	Normal
Cord blood diagnosis	Congenital hypothyroidism	4 (11.8%)	30 (88.2%)
	Normal	4 (0.4%)	974 (99.6%)
Serum sample diagnosis	Congenital hypothyroidism	5 (26.3%)	14 (73.7%)
	Normal	3 (0.3%)	990 (99.7%)

__PRESENT__PRESENT

__PRESENT

## Discussion

CH is a critical condition that is characterized by a deficiency of thyroid hormones, which can lead to severe neurodevelopmental impairments if not diagnosed and treated promptly [2,3]. The incidence varies globally, with studies indicating a prevalence rate ranging from one in 2,000 to one in 4,000 live births [6-8]. Early detection through neonatal screening is crucial to prevent associated morbidities. This study evaluated the efficacy of CBFT4 screening in the presence of metabolic screening and determined the prevalence of CH in a single tertiary center in Saudi Arabia.

The prevalence of CH in our cohort was 0.8%, which was confirmed by NBS, while a higher initial detection rate of 3.4% was obtained based on cord blood screening. The average CBTT4 level was 6.48 ± 0.78 µg/dL, and the average cord blood TSH was 5.9 ± 6.4 µIU/mL. The mean serum FT4 level was 1.3 ± 0.5 ng/dL, and the average serum TSH level was 8.7 ± 24.5 µIU/mL. The analysis of diagnostic accuracy revealed a sensitivity of 50% and a specificity of 97% for cord blood diagnosis, while serum sample diagnosis had a sensitivity of 62.5% and a specificity of 98.6%. Thus, while cord blood screening may have higher initial sensitivity, it also has a higher false positive rate. This discrepancy underscores the importance of confirmatory testing to avoid unnecessary treatment and anxiety for parents [13,19].

Diagnostic accuracy of cord blood screening

Cord blood screening for CH demonstrated a sensitivity of 50% and a specificity of 97%. The high specificity indicates that cord blood screening is reliable for ruling out CH in newborns with normal results. However, the moderate sensitivity highlights a risk of missed diagnoses if cord blood screening is used alone. The PPV of 11.8% and NPV of 99.6% further emphasize that while a normal cord blood result is reassuring, a positive result requires follow-up testing to confirm the diagnosis [12,13].

In comparison, serum sample diagnosis showed slightly higher sensitivity (62.5%) and specificity (98.6%), suggesting that it may be a more accurate method for confirming CH. The higher PPV of 26.3% for serum diagnosis indicates better predictive accuracy for positive results compared to cord blood screening. These findings align with the literature, where serum testing is often considered the gold standard for confirmatory diagnosis due to its higher reliability [19,20].

The overall CH prevalence of 0.8% is consistent with global reports. Sex analysis showed a higher prevalence in females (1.2%) compared to males (0.5%), although this difference was not statistically significant (p = 0.229). This aligns with some studies suggesting a slightly higher incidence in females, although the reasons remain unclear [21,22].

Lower birth weight and gestational age were significantly associated with CH. Newborns with CH had a mean birth weight of 2.146 kg and a mean gestational age of 35 weeks, whereas newborns without CH had a mean birth weight of 2.801 kg (p = 0.006) and gestational age of 37 weeks (p = 0.008). These findings are consistent with previous studies indicating that preterm and low-birth-weight infants are at higher risk for CH, which is likely due to the immaturity of the hypothalamic-pituitary-thyroid axis in these populations [17,18].

Maternal thyroid disease was also associated with an increased risk of CH in newborns. Among mothers who had thyroid disease, 2.5% of their newborns were diagnosed with CH, while only 0.6% of newborns had CH among those without maternal thyroid disease (p = 0.075). Although not statistically significant, this trend supports the evidence that maternal thyroid dysfunction can impact fetal thyroid development [16,18].

Several studies have explored the efficacy of different screening methods for CH. Our findings are in line with research suggesting that while cord blood screening is useful for initial detection, it requires confirmatory testing due to its lower sensitivity and higher false positive rate [19,20]. Serum FT4 and TSH measurements are widely regarded as more accurate for diagnosing CH. Our findings support this, with serum sample diagnosis demonstrating higher sensitivity and specificity than cord blood screening [13,19].

Implications for practice

The findings of this study have important implications for neonatal screening practices in Saudi Arabia and similar settings. Given the moderate sensitivity of cord blood screening, it is crucial to implement robust follow-up protocols to ensure that all positive cases are confirmed through serum testing. This two-tiered approach can help to minimize false positives and ensure timely intervention for true cases of CH. Healthcare providers should also be aware of the increased risk of CH in preterm and low-birth-weight infants and consider more frequent or earlier screening for these populations. Additionally, maternal thyroid disease should prompt closer monitoring of newborn thyroid function, given the potential impact on fetal development.

Strengths and limitations

This study's strengths include its large sample size and comprehensive data collection over a three-year period, which provided a robust dataset for analysis. The use of both cord blood and serum sample testing allowed for a detailed comparison of screening methods and enhanced the validity of the findings. However, there are limitations to consider. The retrospective design may have introduced selection bias as only newborns with available data were included. Additionally, the study was conducted at a single tertiary center, which may have limited the generalizability of the findings to other settings. Future research should include multiple centers and prospective designs to validate these results.

Future directions

Future studies should explore the cost-effectiveness of integrating cord blood screening with follow-up serum testing in routine practice. Additionally, research should examine the underlying mechanisms of CH in preterm and low birth weight infants, which could provide insights for developing targeted screening and intervention strategies. Longitudinal studies should also be done to track neurodevelopmental outcomes in infants diagnosed with CH through different screening methods, which would also be valuable in assessing the long-term impact of early detection and treatment.

## Conclusions

Our study has highlighted the importance of confirmatory testing for CH following initial cord blood screening. While cord blood screening is a useful tool for early detection, its moderate sensitivity necessitates follow-up serum testing to ensure accurate diagnosis. The prevalence of CH in our cohort aligns with global estimates, and the associations with low birth weight, preterm birth, and maternal thyroid disease underscore the need for targeted screening in high-risk groups. Implementing a two-tiered screening approach could improve diagnostic accuracy, ensure timely intervention, and ultimately enhance outcomes for affected infants.

## References

[1] Agrawal P, Philip R, Saran S, Gutch M, Razi MS, Agroiya P, Gupta K (2015). Congenital hypothyroidism. Indian J Endocrinol Metab.

[2] Cherella CE, Wassner AJ (2017). Congenital hypothyroidism: insights into pathogenesis and treatment. Int J Pediatr Endocrinol.

[3] Rose SR, Wassner AJ, Wintergerst KA (2023). Congenital hypothyroidism: screening and management. Pediatrics.

[4] Lauffer P, Zwaveling-Soonawala N, Naafs JC, Boelen A, van Trotsenburg AS (2021). Diagnosis and management of central congenital hypothyroidism. Front Endocrinol (Lausanne).

[5] Shaikh AA, Alsofyani A, Shirah B, Noaim KA, Ahmed ME, Babiker A, Alwan IA (2021). Congenital hypothyroidism in Saudi population in two major cities: a retrospective study on prevalence and therapeutic outcomes. Int J Health Sci (Qassim).

[6] Liu L, He W, Zhu J (2023). Global prevalence of congenital hypothyroidism among neonates from 1969 to 2020: a systematic review and meta-analysis. Eur J Pediatr.

[7] Dayal D, Prasad R (2015). Congenital hypothyroidism: current perspectives. Res Rep Endocr Disord.

[8] Anne RP, Rahiman EA (2022). Congenital hypothyroidism in India: a systematic review and meta-analysis of prevalence, screen positivity rates, and etiology. Lancet Reg Health Southeast Asia.

[9] Boelen A, Zwaveling-Soonawala N, Heijboer AC, van Trotsenburg AS (2023). Neonatal screening for primary and central congenital hypothyroidism: is it time to go Dutch?. Eur Thyroid J.

[10] Olivieri A, Fazzini C, Medda E (2015). Multiple factors influencing the incidence of congenital hypothyroidism detected by neonatal screening. Horm Res Paediatr.

[11] Maggio MC, Ragusa SS, Aronica TS, Granata OM, Gucciardino E, Corsello G (2021). Neonatal screening for congenital hypothyroidism in an Italian Centre: a 5-years real-life retrospective study. Ital J Pediatr.

[12] Al Juraibah F, Alothaim A, Al Eyaid W, AlMutair AN (2019). Cord blood versus heel-stick sampling for measuring thyroid stimulating hormone for newborn screening of congenital hypothyroidism. Ann Saudi Med.

[13] Bhatia R, Rajwaniya D (2018). Congenital hypothyroidism screening in term neonates using umbilical cord blood TSH values. Indian J Endocrinol Metab.

[14] Lau CS, Joseph R, Aw TC (2020). Screening for congenital hypothyroidism. Ann Acad Med Singap.

[15] Ahmad A (2024). Evaluating neonatal screening methods for congenital hypothyroidism: a comparative analysis of cord blood and venous blood sampling. J Heart Valve Dis.

[16] Al-Agha A, Alghalbi D, Ashgar S (2016). Clinical presentations of congenital hypothyroidism and associated abnormalities in children treated at Western Saudi Arabia. Res Rev J Med Health Sci.

[17] Hashemipour M, Samei P, Kelishadi R (2019). A systematic review on the risk factors of congenital hypothyroidism. J Pediatr Rev.

[18] Khammarnia M, Siakhulak FR, Ansari H, Peyvand M (2018). Risk factors associated with congenital hypothyroidism: a case-control study in southeast Iran. Electron Physician.

[19] Wong SL, Jalaludin MY, Zaini AA (2015). Congenital hypothyroidism: an audit and study of different cord blood screening TSH values in a tertiary medical centre in Malaysia. Adv Endocrinol.

[20] Shankar M, Chaudhary R, Chaudhary AK (2019). Use of umbilical cord blood TSH as a marker for screening of congenital hypothyroidism in a tertiary care centre in Jharkhand. IOSR J Dent Med Sci.

[21] Tuli G, Munarin J, Tessaris D, Matarazzo P, Einaudi S, de Sanctis L (2021). Incidence of primary congenital hypothyroidism and relationship between diagnostic categories and associated malformations. Endocrine.

[22] Deng K, He C, Zhu J (2018). Incidence of congenital hypothyroidism in China: data from the national newborn screening program, 2013-2015. J Pediatr Endocrinol Metab.

